# Early versus late awake prone positioning in non-intubated patients with COVID-19

**DOI:** 10.1186/s13054-021-03761-9

**Published:** 2021-09-17

**Authors:** Ramandeep Kaur, David L. Vines, Sara Mirza, Ahmad Elshafei, Julie A. Jackson, Lauren J. Harnois, Tyler Weiss, J. Brady Scott, Matthew W. Trump, Idrees Mogri, Flor Cerda, Amnah A. Alolaiwat, Amanda R. Miller, Andrew M. Klein, Trevor W. Oetting, Lindsey Morris, Scott Heckart, Lindsay Capouch, Hangyong He, Jie Li

**Affiliations:** 1 Division of Respiratory Care, Department of Cardiopulmonary Sciences, Rush University Medical Center, 600 S Paulina St, Suite 765, Chicago, IL USA; 2 Division of Pulmonary, Critical Care, and Sleep Medicine, Rush University Medical Center, Chicago, IL USA; 3 Department of Respiratory Care, Unity Point Health-Des Moines, Des Moines, IA USA; 4 The Iowa Clinic P.C. and Unity Point Health-Des Moines, Des Moines, IA USA; 5 Pulmonary and Critical Care Medicine Division, Texas A&M School of Medicine, Baylor University Medical Center, Dallas, TX USA; 6 Nursing, MICU, Rush University Medical Center, Chicago, IL USA; 7 Department of Respiratory and Critical Care Medicine, Beijing Institute of Respiratory Medicine, Beijing Chao-Yang Hospital, Capital Medical University, Beijing, China

**Keywords:** Awake prone positioning, Non-intubated, COVID-19, Coronavirus, Acute hypoxemic respiratory failure

## Abstract

**Background:**

Awake prone positioning (APP) is widely used in the management of patients with coronavirus disease (COVID-19). The primary objective of this study was to compare the outcome of COVID-19 patients who received early versus late APP.

**Methods:**

Post hoc analysis of data collected for a randomized controlled trial (ClinicalTrials.gov NCT04325906). Adult patients with acute hypoxemic respiratory failure secondary to COVID-19 who received APP for at least one hour were included. Early prone positioning was defined as APP initiated within 24 h of high-flow nasal cannula (HFNC) start. Primary outcomes were 28-day mortality and intubation rate.

**Results:**

We included 125 patients (79 male) with a mean age of 62 years. Of them, 92 (73.6%) received early APP and 33 (26.4%) received late APP. Median time from HFNC initiation to APP was 2.25 (0.8–12.82) vs 36.35 (30.2–75.23) hours in the early and late APP group (*p* < 0.0001), respectively. Average APP duration was 5.07 (2.0–9.05) and 3.0 (1.09–5.64) hours per day in early and late APP group (*p* < 0.0001), respectively. The early APP group had lower mortality compared to the late APP group (26% vs 45%, *p* = 0.039), but no difference was found in intubation rate. Advanced age (OR 1.12 [95% CI 1.0–1.95], *p* = 0.001), intubation (OR 10.65 [95% CI 2.77–40.91], *p* = 0.001), longer time to initiate APP (OR 1.02 [95% CI 1.0–1.04], *p* = 0.047) and hydrocortisone use (OR 6.2 [95% CI 1.23–31.1], *p* = 0.027) were associated with increased mortality.

**Conclusions:**

Early initiation (< 24 h of HFNC use) of APP in acute hypoxemic respiratory failure secondary to COVID-19 improves 28-day survival.

*Trial registration* ClinicalTrials.gov NCT04325906.

## Introduction

Coronavirus disease (COVID-19) is a viral infectious disease caused by coronavirus (SARS-CoV-2) [1]. COVID-19 primarily affects the respiratory system causing mild to severe respiratory illness. Around 25–30% of COVID-19 patients develop signs of acute respiratory distress requiring higher respiratory support in terms of oxygen therapy, noninvasive and invasive positive pressure ventilation [2]. Prone positioning improves oxygenation by the uniform distribution of tidal volume and recruitment of the dorsal lung regions leading to improved lung compliance [3]. Before the COVID-19 pandemic, a small, prospective observational study demonstrated the benefit of using prone positioning among non-intubated patients with moderate acute respiratory distress syndrome (ARDS) to reduce the need for invasive mechanical ventilation [4]. Since the emergence of COVID-19, this technique has been extensively used to improve oxygenation in non-intubated COVID-19 patients with acute hypoxemic respiratory failure (AHRF) [5].

There is evidence demonstrating the benefits of early prone positioning to improve oxygenation and patient outcomes in intubated patients with moderate to severe ARDS [6]. A recent multicenter cohort study investigating the timing of prone positioning initiation among mechanically ventilated patients with COVID-19 found a lower hospital mortality among those who received early prone positioning (within 2 days of ICU admission) [7]. Another retrospective, multicenter observational study included 827 non-intubated patients with COVID-19 and found that awake prone positioning (APP) was significantly associated with lower mortality (20.0% vs 37.9%; *p* < 0.0001) and intubation rate (23.6% vs 40.4%; *p* < 0.0001) as compared to supine position [8]. Randomized controlled trials have been done to evaluate the feasibility of implementation and patient compliance with APP in patients with COVID-19, but no long-term outcomes were assessed [9–13]. A recent systematic review found that APP improved oxygenation among patients with AHRF due to COVID-19, however, APP did not reduce intubation rates [14]. Finally, a collaborative meta-trial of six randomized controlled superiority trials, on which this post hoc analysis is based, enrolled a total of 1121 patients and found hazard ratios of 0.75 (95% CI, 0.62–0.91) for intubation and 0.87 (95% CI, 0.68–1.11) for 28-day mortality with APP, as compared to the standard care group [15].

Despite multiple studies showing benefit of prone positioning among non-intubated patients with COVID-19, there is no clear evidence available guiding the timing of awake prone positioning for patients with COVID-19 to achieve optimal patient outcomes [16]. Therefore, the primary objective of this study was to compare early versus late initiation of awake prone positioning (APP) on patient outcomes, including hospital mortality and the need for invasive mechanical ventilation (IMV).

## Methods

From April 2nd, 2020 to January 26th, 2021, we participated in a collaborative meta-trial of six randomized controlled open-label superiority trials [17] to compare the effectiveness of APP versus standard care in patients with AHRF due to COVID-19 supported with high-flow nasal cannula (HFNC). The intent of the unique study design was to achieve results with a sufficient effect size, in a faster manner, and at a lower cost [18]. It was felt this novel approach was necessary during a global event, like the COVID-19 pandemic. Four hospitals joined in the American trial (NCT04325906) and the randomization was assigned by the leading institution (Rush University Medical Center). The study protocol was approved by the institutional review board (20032604-IRB01). This post hoc analysis was conducted using the American data set.

### Study procedure

In the American trial, patients were enrolled to receive APP or standard care if they were diagnosed with AHRF secondary to COVID-19 and had the ratio of saturation of pulse oximetry (SpO_2_) to the fraction of inspired oxygen (FiO_2_) < 240. All patients received respiratory support via high-flow nasal cannula (HFNC) initiated at 50 L/min with FiO_2_ titrated to maintain SpO_2_ between 90 and 95%. HFNC was discontinued when the weaning criteria of F_i_O_2_ at 0.4 and flow at 40 L/min were met. For the patients who were randomized to the APP group, prone positioning was performed under clinician supervision and patients were instructed to maintain prone positioning as long as tolerated. In the standard care group, prone positioning was discouraged and if occurred, it was recorded as a protocol violation.

In this post hoc analysis, patients who received APP for a minimum of one hour were included, regardless of the group (APP or standard care) they were originally assigned. The subjects were excluded if the information on APP was missing.

### Data collection

For the original study, demographic and clinical data were attained from the patient’s electronic medical record. Demographic data included age, gender, ethnicity, height, and weight. Clinical data included medical history, medications list, laboratory, and microbiology findings. Data related to APP included vital signs and HFNC settings before and after the first APP session and start/end time for each prone session in the first three days of APP. ROX index was calculated using SpO_2_/FiO_2_ divided by respiratory rate [19]. The types of respiratory interventions including noninvasive and invasive positive pressure ventilation, use of inhaled vasodilator via HFNC or invasive ventilation, and the need for extracorporeal membrane oxygenation (ECMO) during hospitalization were recorded. Length of intensive care unit (ICU) and hospital stay, as well as the hospital outcome, were obtained.

### Definitions and study outcome

Early APP was defined as APP initiated within 24 h of starting HFNC therapy. The primary study outcomes were 28-day mortality and intubation rate among patients that received early vs late APP.

### Statistical analysis

Continuous variables are presented as means ± standard deviation (SD or as medians and interquartile ranges (IQR). Comparison of continuous variables between early vs late APP groups was conducted using Student’s t test for variables with a normal distribution and using the Mann–Whitney U test for variables with a non-normal distribution. Results with respect to categorical variables are presented as proportions and were analyzed with chi-square or Fisher's exact tests. A multivariate logistic regression model was used to identify the risk factors for hospital death. Outcome variables and covariates were assessed using the enter method, model fit was assessed by Hosmer–Lemeshow goodness-of-fit statistic and model performance by the classification tables. Statistically significant independent variables were maintained in the model. Kaplan–Meier method was used to perform the survival analysis between the two study groups. All reported p values are two sided and a *p* value < 0.05 was considered significant. Statistical analysis was done using SPSS 26.0 for Windows (SPSS, Inc., Chicago, IL, USA).

## Results

### Subject characteristics

**Fig. 1:**
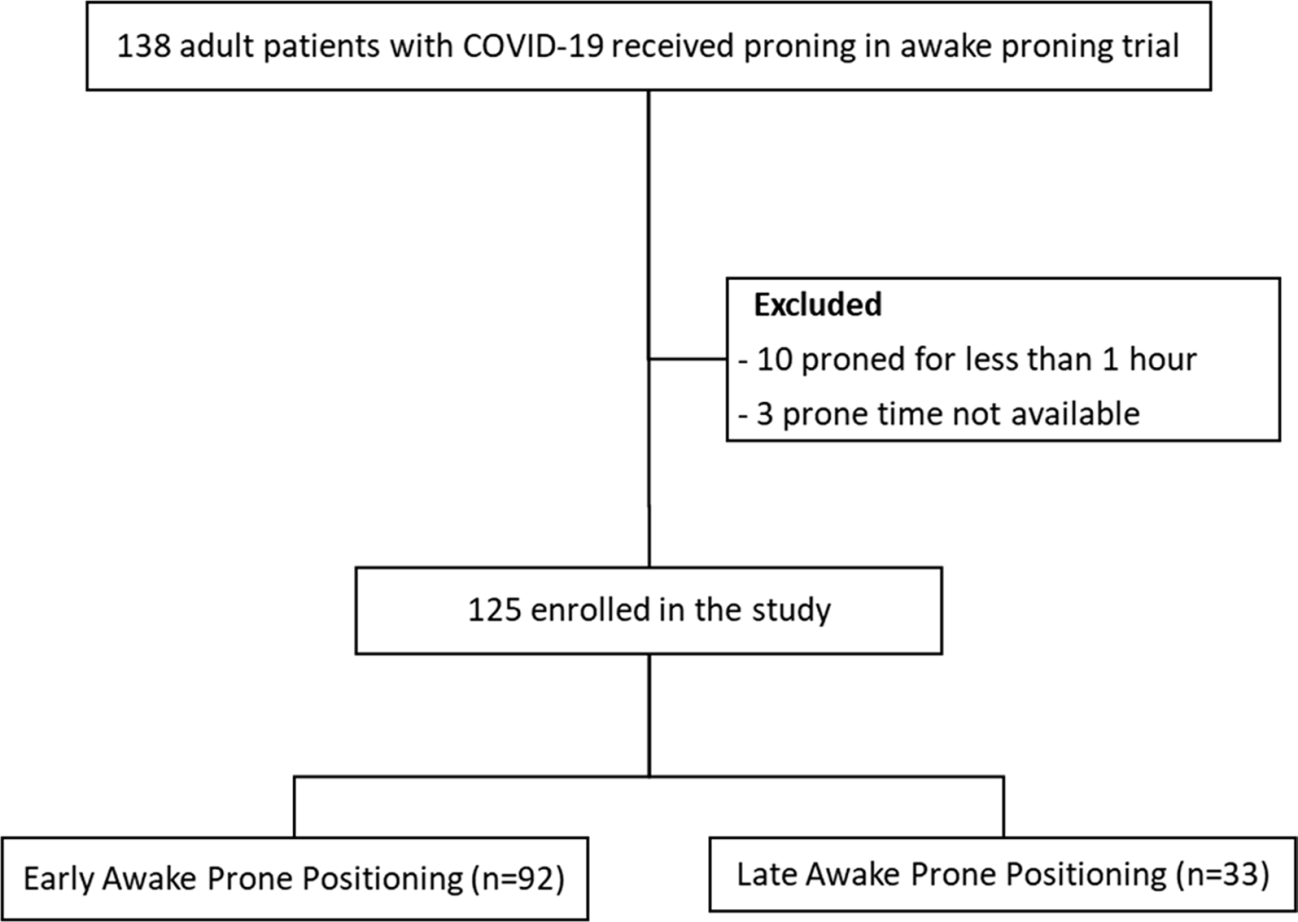
Study flow diagram

Between April 2nd, 2020 and January 26th, 2021, 222 patients with COVID-19 were enrolled in the American trial, 112 patients were assigned to receive APP and 110 patients were assigned to standard care, of whom 26 patients had protocol violation to receive rescue APP. Thus, 138 patients (112 from APP group and 26 from control group) were included for the post hoc analysis (Fig. 1). Of these 138 patients, 13 were excluded due to being self-proned for less than one hour (*n* = 10) and missing data (n=3). A total of 125 patients were enrolled in this study, of whom 92 (73.6%) received early APP and 33 (26.4%) received late APP. Overall, the mean age of those included in this study was 62 (± 11.9) years, 79 (63.2%) patients were males and 70 (56%) were of Hispanic/Latino ethnicity. The median sequential organ failure assessment (SOFA) score was 3 (IQR 2–4.5) at study enrollment for all patients included in this study. The median HFNC set flow was 50 (IQR 50–60) L/min and FiO_2_ was 0.6 (IQR 0.5–0.75) at study enrollment. Demographic and baseline clinical characteristics were similar in both groups (Table 1), except for fewer Hispanic patients (33.3% vs 64.1%, *p* = 0.002) and more Caucasian patients (45.5% vs 23.9%, *p* = 0.027) in the late prone group.

**Table 1:** Overall subject baseline characteristics and comparison between the early and late awake prone positioning group

Variables	Overall (*n* = 125)	Early awake prone (*n* = 92)	Late awake prone (*n* = 33)	*P* value
Age, mean (SD)	62.0 ± 11.9	61.1 ± 12.3	64.9 ± 10.4	0.113
Male, *n* (%)	79 (63.2)	56 (61)	23 (67)	0.367
BMI (kg/m^2^), mean (SD)	30 ± 5.0	30.23 ± 4.96	29.47 ± 5.14	0.411
*Ethnicity, n (%)*				
Hispanic/Latino	70 (56)	59 (64.1)	11 (33.3)	0.002
Caucasian	37 (29.6)	22 (23.9)	15 (45.5)	0.027
African American	7 (5.6)	5 (5.4)	2 (6.1)	0.59
Asian	4 (3.2)	2 (2.2)	2 (6.1)	0.28
Unknown	3 (2.4)	1(1.1)	2 (6.1)	0.17
Others	4 (3.2)	3 (3.3)	1 (3)	0.60
*Comorbidities, n (%)*				
Diabetes Mellitus	54 (43.2)	17 (51.5)	37 (40.2)	0.261
Chronic Lung Disease	17 (13.6)	10 (11)	7 (21)	0.119
Cardiovascular Disease	29 (23.2)	18 (19.6)	11 (33.3)	0.108
Chronic Renal Disease	11 (8.8)	10 (10.9)	1 (3)	0.157
Chronic Liver Disease	1 (0.8)	1 (1.1)	0	0.736
Immunocompromised Condition	16 (12.8)	11 (12)	5 (15)	0.637
Neurologic disease	5 (4)	4 (4.3)	1 (3)	0.603
Others	25 (20)	23 (25)	2 (6.1)	0.020
*Smoking status, n (%)*				0.12
Current Smoker	4 (3.2)	4 (4.3)	0	
Former Smoker	38 (30.4)	23 (25)	15 (45.5)	
Never	73 (58.4)	57 (62)	16 (48.5)	
Not available	10 (8)	8 (8.7)	2 (6)	
SOFA score on admission, median (IQR)	3 (2–4.5)	3 (2–4.75)	3 (3–4.5)	0.70
Assigned to APP group, *n* (%)	101 (80.8)	88 (96)	13 (39)	
SpO_2_/FiO_2_ ratio on enrollment, median (IQR)	143.8 (117.5–174.4)	135 (116.2–166.5)	155 (131.6–188.5)	0.052
Time from hospital admission to APP start (h), median (IQR)	27.48 (13.1–64.2)	18 (7.1–43.2)	60 (34.9–105)	<0.001
Time from HFNC start to APP (h), median (IQR)	8.58 (1.31–24.87)	2.25 (0.8–12.82)	36.35 (30.2–75.23)	<0.001
Total APP hours in the first three days, median (IQR)	13.08 (3.5–43.25)	16 (5.4–51.5)	5 (2.5–17.5)	0.004
APP hours/day, median (IQR)	4.45 (1.75–8.37)	5.07 (2–9.05)	3 (1.09–5.64)	0.006
HFNC duration (d), median (IQR)	6 (2.97–9.46)	5 (2.2–9)	6 (3.2–10.5)	0.18
Antiviral therapy, *n* (%)	84 (67.2)	65 (70.7)	19 (57.6)	0.12
Steroids use, *n* (%)	93 (74.4)	64 (69.6)	29 (87.9)	0.039
Time from HFNC start to steroid start (h), median (IQR)	− 12.48 (− 25.3 to 4.58)	− 14.47 (− 33 to 0)	− 8.57 (− 20.8 to 7. 93)	0.19
*Steroids type, n (%)*				
Dexamethasone	82 (65.6)	56 (60.9)	26 (78.8)	0.063
Hydrocortisone	18 (14.4)	13 (14.1)	5 (15.1)	0.54
Methylprednisone/Prednisone	15 (12)	10 (10.9)	5 (15.1)	0.35

*SD* standard deviation, *BMI* body mass index, *SOFA* sequential organ failure assessment, *IQR* interquartile range, *SpO*_*2*_ saturation of pulse oximetry, *FiO*_*2*_ fraction of inspired oxygen, *HFNC* High-Flow Nasal Cannula, *APP* Awake Prone Positioning

### Time from HFNC initiation to APP

Overall, 67 (54%) patients received APP within 11 h of HFNC initiation, 25 (20%) within 12–23 h, 16 (13%) within 24–35 h and 17 (14%) after 36 h of initiating HFNC. The median time to start APP after initiating HFNC was 2.25 h in early APP group and 36.35 h among those who received late APP (*p* < 0.001) (Table 1). The median time to start APP from hospital admission was 18 h in the early and 60 h in the late APP group (*p* < 0.001). The early APP group spent a median of 5.07 h/day and the late group spent a median of 3 h/day in the prone position. (*p* = 0.006).

### Oxygenation response

**Fig. 2:**
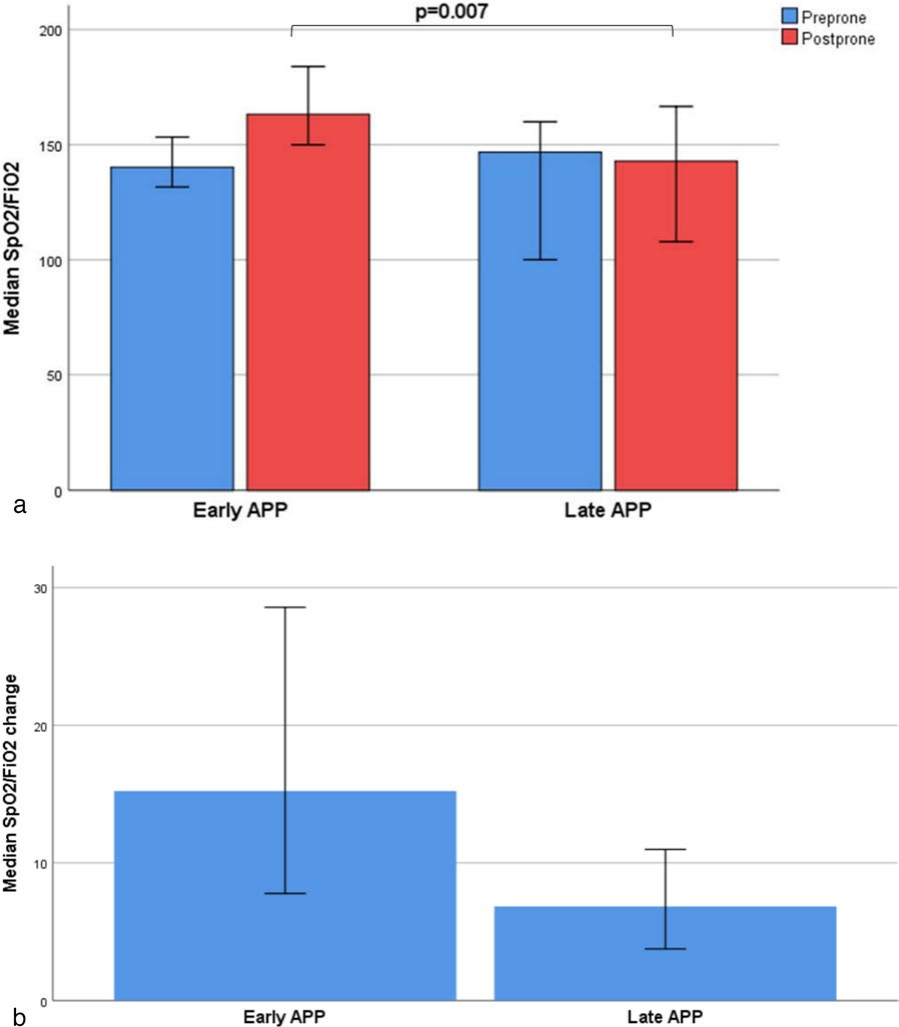
Oxygenation response assessment using SpO_2_/FiO_2_ during first prone session. SpO_2_/FiO_2_ values were recorded 5 min before and 30 min after APP. SpO_2_/FiO_2_ values are presented as median with 95% confidence interval. (APP, awake prone positioning; SpO_2_, saturation of pulse oximetry; FiO_2_, fraction of inspired oxygen)

**Fig. 3:**
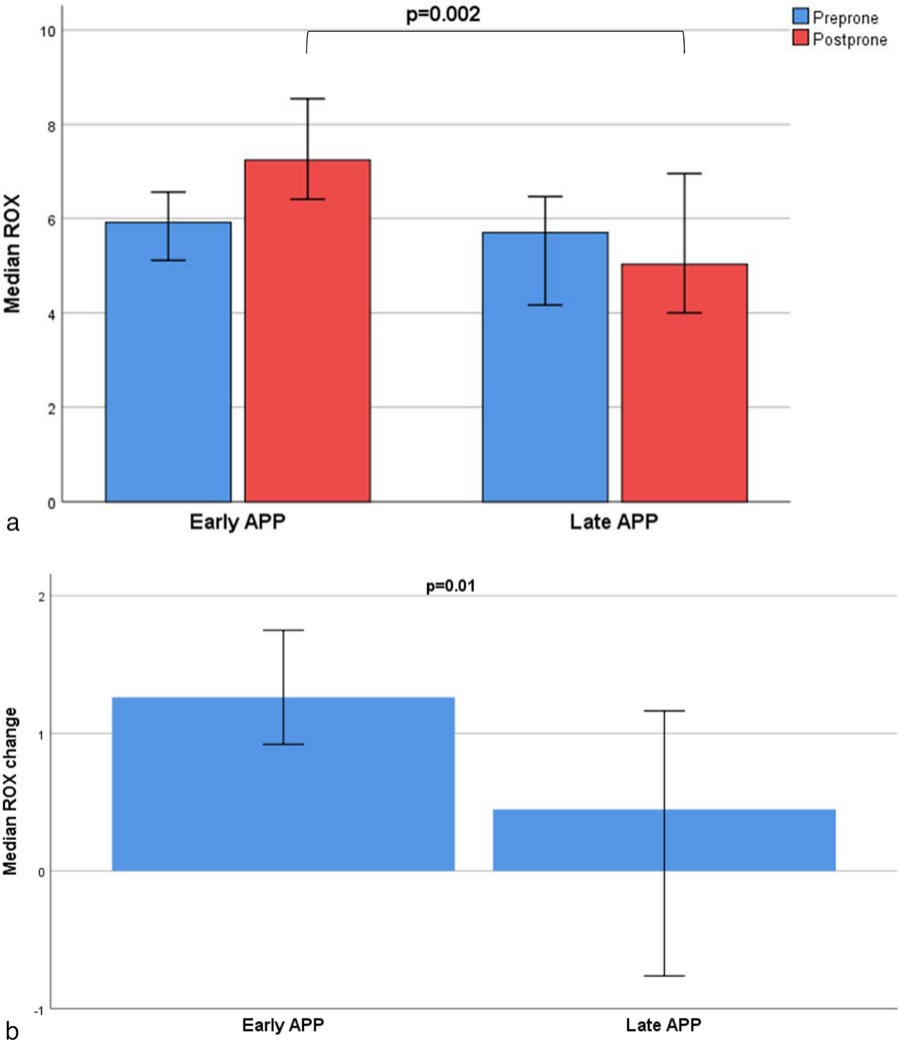
Oxygenation Response Assessment using ROX index during first prone session. ROX was recorded 5 min before and 30 min after APP. ROX index values are presented as median with 95% confidence interval. (APP, awake prone positioning; ROX index represents SpO_2_/FiO_2_ ratio divided by respiratory rate)

There was no significant difference in the SpO_2_/FiO_2_ ratio or ROX index before APP in the early vs late APP groups; however, after 30 min in the first prone session, the early APP group had a higher SpO_2_/FiO_2_ ratio [163.2 (132.8–211) vs 141.4 (105–172.5); *p* = 0.007] (Fig. 2a) and ROX index [7.24 (5–9.93) vs 5 (3.8–6.95); *p* = 0.002] (Fig.  3a). There was no significant difference in SpO_2_/FiO_2_ ratio change [15.22 (3.94–44) vs 6.83 (2.1–19.24); *p* = 0.076] (Fig. 2b) between the two groups but the ROX index change was lower in the late APP group [(1.26 (0.31–2.89) vs 0.21 (−1.57 to 1.1); *p* = 0.01] (Fig. 3b).

### Study outcome

**Fig. 4:**
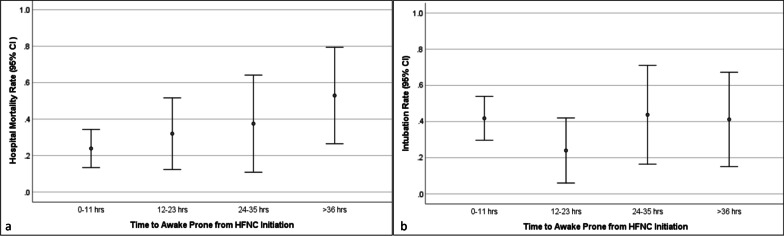
Time to initiation of awake prone positioning and patient outcome (HFNC, high-flow nasal cannula; CI, confidence interval)

**Fig. 5:**
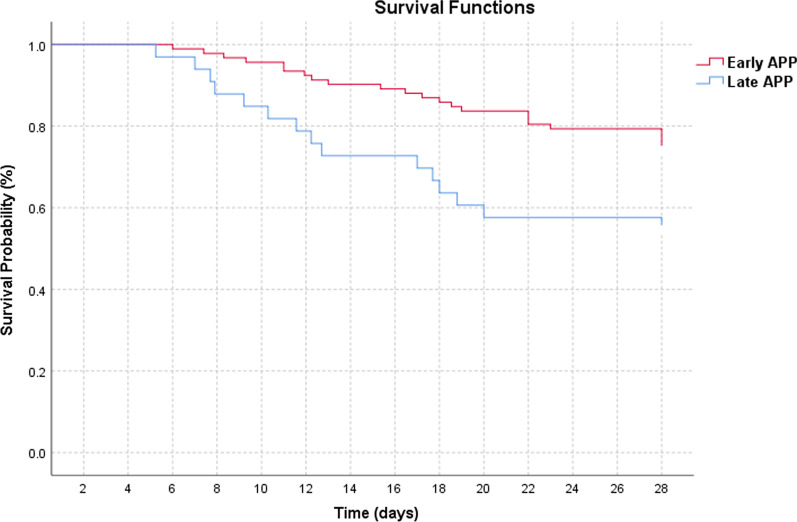
Kaplan–Meier Survival probabilities over 28 days after hospital admission (APP, awake prone positioning)

As the duration between HFNC initiation to APP increased, the hospital mortality increased (Fig. 4a) with no impact on the intubation rate (Fig. 4b). The late APP group had a significantly higher mortality as compared to those who received early APP (45% vs 26%, *p* = 0.039; Table 2). The Kaplan–Meier survival plot demonstrated a decrease in survival among patients who received late APP (Fig. 5). The hospital and ICU length of stay were similar for the two groups. In terms of intubation rate and IMV duration, 48 (38.4%) patients were intubated overall and there was no significant difference in intubation rate and IMV duration among the two study groups. The average time from HFNC initiation to intubation was similar in both groups with median time to intubation of 5.13 (1.89–10.85) days in the early APP group and 5.27 (3.2–9.56) days in the late APP group (*p* = 0.65). Similarly, there was no difference in the two groups in terms of time from APP start to intubation. NIV use and duration from HFNC initiation to NIV were also similar among the two study groups. The use of rescue respiratory support such as ECMO and inhaled vasodilators was similar. There was no significant difference among the study groups in terms of antiviral therapy (remdesivir), however, more patients received steroids in the late APP group (87.9% vs 66.3%, *p* = 0.039).

**Table 2:** Study outcome comparison between the early and late awake prone group

Outcomes	Early Awake Prone (*n* = 92)	Late Awake Prone (*n* = 33)	*P* value
28 day mortality, *n* (%)	24 (26)	15 (45)	0.039
Death without intubation, * n* (%)	7 (7.6)	6 (18.2)	0.088
Hospital LOS (d), median (IQR)	13.97 (9.64–24.9)	12.53 (9–20.9)	0.66
ICU LOS (d), median (IQR)	7.91 (4.25–21)	8 (3.38–16.9)	0.55
IMV use, * n* (%)	34 (37)	14 (42.4)	0.58
IMV duration (d), median (IQR)	10.59 ± 6.12	8.89 ± 6.10	0.43
Time from HFNC start to intubation (d), median (IQR)	5.13 (1.89–10.85)	5.27 (3.2–9.56)	0.65
Time from APP start to intubation (d), median (IQR)	4.73 (1.85–10.6)	3.12 (1.31–8.23)	0.37
NIV use, * n* (%)	23 (25)	5 (15.2)	0.24
Time from HFNC start to NIV (d), median (IQR)	3.74 (0.83–9.61)	2.11 (0.7–8.95)	0.77
ECMO use, * n* (%)	2 (2.2)	0	0.54
Inhaled vasodilator use, * n* (%)	26 (28.3)	9 (27.3)	0.28

*LOS* length of stay, *IQR* interquartile range, *ICU* intensive care unit, *IMV* invasive mechanical ventilation, *NIV* noninvasive ventilation, *HFNC* high-flow nasal cannula, *ECMO* extracorporeal membrane oxygenation, *APP* Awake Prone Positioning

### Risk factors associated with hospital death

In the multivariate logistic regression analysis, factors independently associated with hospital mortality were advanced age (OR 1.12 [95% CI 1–1.95], *p* = 0.001), use of invasive mechanical ventilation (OR 10.65 [95% CI 2.77–40.91], *p* = 0.001), longer time to initiate prone from HFNC start (OR 1.02 [95% CI 1–1.04], *p* = 0.047) and hydrocortisone use (OR 6.2 [95% CI 1.23–31.1], *p* = 0.027).

## Discussion

To our knowledge, this is the first study to explore the association between the time to initiate APP (early vs late) and mortality among non-intubated patients with COVID-19. In this post hoc analysis, we found that late APP was independently associated with an increase in hospital mortality, however, the association between exposure and outcome is weak (OR 1.02 [95% CI 1–1.04]). Patients who were proned within 24 h of initiating HFNC for AHRF (early APP) had a lower mortality than those who received APP after 24 h (late APP). Additionally, being elderly, intubated, and receiving hydrocortisone were significantly associated with mortality at 28 days.

Awake prone positioning is a noninvasive technique widely used among patients with COVID-19 and was found to reduce the need for invasive mechanical ventilation and improve patient outcomes in the recent large, meta-trial of six randomized controlled open-label superiority trials conducted across six countries [15]. The present study results suggest that initiating early prone positioning within 24 h of HFNC start further improves the 28-day mortality.

Several reasons may explain our research findings. First, COVID-19-induced lung injury is described to have two distinctive phenotypes, type L and type H [20]. Type L represents high compliance/low elastance, and low alveolar recruitability, while type H represents low compliance/high elastance and high alveolar recruitability [21]. Type H reportedly responds well to positive end expiratory pressure and prone positioning. This phenotype likely represents a time-related disease spectrum where the early use of HFNC and APP may improve ventilation-perfusion matching and reduce dyspnea related work of breathing and patient self-inflicted lung injury [22, 23]. High respiratory drive, commonly found among patients with COVID-19, is associated with increased transpulmonary pressure changes that lead to vascular leakage and self-inflicted lung injury in patients with existing lung injury [24]. Furthermore, respiratory rates exceeding 22 breaths/minutes are associated with a 1.9–3.2 fold increase in mortality risk in patients with COVID-19 [25]. In the present study, we found that early use of APP led to significant improvements in the oxygenation status and work of breathing, assessed by SpO_2_/FiO_2_ ratio and ROX index. Patients who received early APP had significantly higher SpO_2_/FiO_2_ ratios after being proned for 30 min as compared to those who received late prone positioning. Similarly, the ROX index, a predictor of outcome with the use of HFNC among patients with acute respiratory failure [19], was higher among patients that received early APP.

Based on morphology, early ARDS represents the exudative phase with diffuse alveolar damage, interstitial and alveolar damage followed by a late, fibroproliferative phase with interstitial fibrosis [26]. Among mechanically ventilated ARDS patients, Nakos et al. reported that patients with early ARDS (≤ 36 h from onset) had a superior oxygenation response after prone positioning than those with late ARDS (> 36 h from onset) [27]. Similarly, in a prospective study by Coppo et al., the investigators reported an average time between hospital admission to prone positioning of 2.7 days among those who responded to prone positioning, compared to 4.6 days among non-responders in non-intubated patients with COVID-19 [28]. However, their study did not report mortality between the groups. In our study, early APP group received proning within 0.75 days and late APP within 2.5 days of hospital admission. Thus, utilization of early APP during the exudative/inflammatory phase may have led to a better oxygenation response, as well as improvement in work of breathing, which may translate into improved hospital survival.

Second, patients in the early APP group were in the prone position for more total hours than those in the late APP group. On average, the early APP group received 5.07 h/day of prone positioning as compared to 3 h/day in the late APP group (*p* = 0.006). Prior studies have demonstrated the mortality benefit of using a longer duration of prone session among intubated patients [3].

Third, a significantly lower number of patients in the early APP group received steroids (69.6% vs 87.9%; *p* = 0.03). The use of steroids is widely reported for clinical management of severe COVID-19 [29]. In one retrospective, observational study steroid use is associated with increased mortality among patients with COVID-19 [30]. In a meta-analysis, the authors reported some beneficial effects of corticosteroids, but note that the overall mortality was higher for those patients that received corticosteroids [31]. A recent open-label randomized controlled (RECOVERY) trial demonstrated a mortality benefit when dexamethasone was used for patients with COVID-19 patients receiving oxygen or IMV [32]. However, another randomized controlled trial evaluating the effectiveness of hydrocortisone in COVID-related ARDS demonstrated no significant reduction in mortality or continued use of respiratory support [33]. In our multivariate analysis, we found that hydrocortisone use, alone or in addition to dexamethasone or prednisone/methylprednisone, was independently associated with the increased 28-day mortality. This might be explained by the likelihood that sicker patients may have received hydrocortisone. Previous studies have demonstrated the increased use of corticosteroids among COVID-19 patients with shock [7]. These study findings suggest further exploration of hydrocortisone use in the treatment of COVID-19 is needed.

This study has several limitations. First, this study is a post hoc analysis of the data collected for a previous randomized clinical trial. Even though post hoc analyses are inherently flawed (due to the lack of randomization, inflated statistical significance, etc.), they do provide exploratory information that could be used to generate hypotheses for future studies [34, 35]. Due to the post hoc nature of this study, we were unable to calculate the sample size to detect the true difference. The sample size in this study is very small which may have impacted the magnitude of an association between the study groups and outcomes [36]. This study included patients from only three hospitals in the USA that may have different clinical management strategies for COVID-19 patients as compared to other clinical settings. Additonally, the original study did not record computed tomography findings to further explore the underlying pathophysiological features associated with increased mortality in the late APP group. Finally, the original study did not collect the incidence of shock or daily disease severity scores that may have prompted the use of steroids such as hydrocortisone among the critically ill patients.

## Conclusion

For patients with acute hypoxemic respiratory failure secondary to COVID-19 and require HFNC therapy, early awake prone positioning (< 24 h of HFNC use) is associated with lower 28-day mortality.

## Data Availability

Any data related question should be directed to the corresponding author.

## Abbreviations

APP: Awake prone positioning
ARDS: Acute respiratory distress syndrome
AHRF: Acute hypoxemic respiratory failure
CDC: Centers for Disease Control and Prevention
CI: Confidence interval
COVID-19: Coronavirus disease
ECMO: Extracorporeal membrane oxygenation
HFNC: High-flow nasal cannula
IMV: Invasive mechanical ventilation
OR: Odds ratio
SpO_2_: Saturation of pulse oximetry
FiO_2_: Fraction of inspired oxygen
ICU: Intensive care unit
NIV: Noninvasive ventilation
